# Influence of Cations on HCOOH and CO Formation during CO_2_ Reduction on a Pd_ML_Pt(111) Electrode

**DOI:** 10.1021/jacs.3c03786

**Published:** 2023-08-31

**Authors:** Chunmiao Ye, Federico Dattila, Xiaoting Chen, Núria López, Marc T. M. Koper

**Affiliations:** † Leiden Institute of Chemistry, Leiden University, 2300 RA Leiden, The Netherlands; ‡ Institute of Chemical Research of Catalonia (ICIQ-CERCA), The Barcelona Institute of Science and Technology (BIST), 43007 Tarragona, Spain

## Abstract

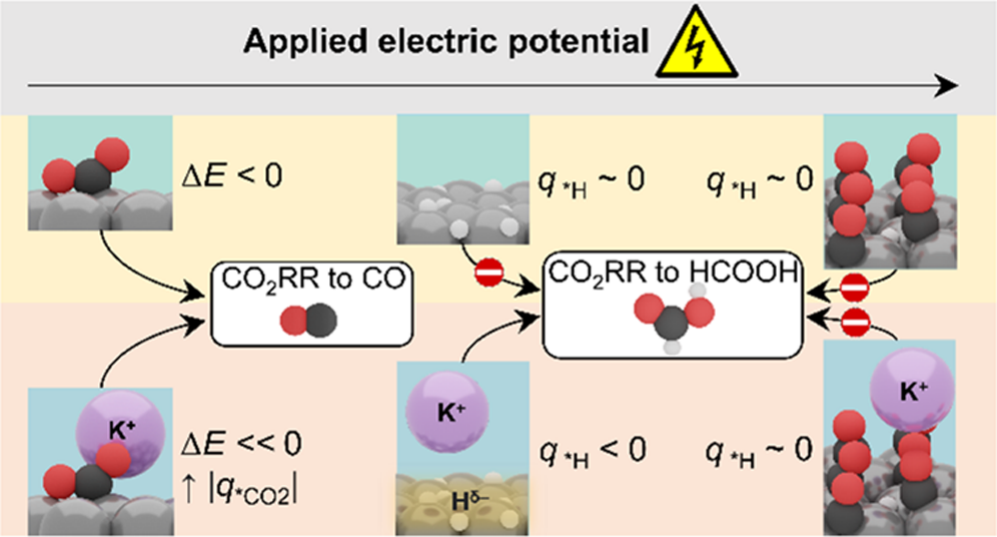

Understanding the role of cations in the electrochemical CO_2_ reduction (CO_2_RR) process is of fundamental importance for practical application. In this work, we investigate how cations influence HCOOH and CO formation on Pd_ML_Pt(111) in pH 3 electrolytes. While only (a small amount of adsorbed) CO forms on Pd_ML_Pt(111) in the absence of metal cations, the onset potential of HCOOH and CO decreases with increasing cation concentrations. The cation effect is stronger on HCOOH formation than that on CO formation on Pd_ML_Pt(111). Density functional theory simulations indicate that cations facilitate both hydride formation and CO_2_ activation by polarizing the electronic density at the surface and stabilizing *CO_2_^–^. Although the upshift of the metal work function caused by high coverage of adsorbates limits hydride formation, the cation-induced electric field counterbalances this effect in the case of *H species, sustaining HCOOH production at mild negative potentials. Instead, at the high *CO coverages observed at very negative potentials, surface hydrides do not form, preventing the HCOOH route both in the absence and presence of cations. Our results open the way for a consistent evaluation of cationic electrolyte effects on both activity and selectivity in CO_2_RR on Pd–Pt catalysts.

## Introduction

The electrochemical carbon dioxide reduction reaction (CO_2_RR) into fuels and chemicals has recently gained substantial attention as a promising method to utilize abundant carbon feedstock and to store excess renewable electric energy. In aqueous media, the CO_2_RR can generate various products, including formic acid (HCOOH), carbon monoxide (CO), hydrocarbons, and alcohols, depending on the catalyst and the microenvironment.^1−4^ Therefore, understanding the factors determining selectivity at the molecular level is crucial to ensure the future application of CO_2_RR.

The nature of the metal catalyst has an important effect on the activity and selectivity in CO_2_RR. Of the monometallic catalysts, Cu exhibits a unique selectivity to methane and various multi-carbon products such as ethylene, ethanol, and propanol.^5−8^ Precious metal catalysts such as Au and Ag show high selectivity toward CO in CO_2_RR.^4,9^ Platinum group metals are also selective for CO formation but are affected by CO surface poisoning.^3,10−12^ Pd-based catalysts can reach a Faradaic efficiency of 95% for CO generation at negative potentials (−0.8 V_RHE_).^10,11^ However, this selectivity is potential dependent, and Pd can also deliver a high Faradaic efficiency toward HCOOH (close to 99%) with a small overpotential of 0.1 V.^3^ Although Pd deactivates over a few hours because of the simultaneous formation of (adsorbed) CO,^3^ Pd is an excellent candidate to study the electrochemical CO_2_RR because of this selectivity switch between HCOOH and CO formation in different potential windows.

To better understand the special reactivity of Pd for CO_2_RR, detailed investigations on atomically well-defined Pd surfaces are highly desirable. Pt single crystals modified with a Pd monolayer are good candidates as their electrochemical and electrocatalytic properties have been shown to be similar to bulk Pd single crystals, but they do not exhibit the bulk-like hydrogen absorption of Pd crystals (a process that will mask the CO_2_RR current).^13−15^ Recent work has shown that a Pd_ML_Pt(111) single-crystal electrode has a high, almost reversible catalytic activity for the reduction of CO_2_ to HCOOH and the oxidation of HCOOH to CO_2_, very similar to bulk Pd crystals.^16^ The high activity for the reduction of CO_2_ to HCOOH at a low overpotential was ascribed to the hydridic (negatively charged) character of the surface-adsorbed hydrogen *H^δ−^ on Pd_ML_Pt(111), which reacts with CO_2_ in the solution close to the metal to form HCOOH.^3,16^ This hydridic character was reported to depend on the metal work function.^3,16^ At more negative potentials, *CO_2_ binding through carbon becomes more favorable, eventually leading to the formation of surface-adsorbed *CO, which poisons the electrode.^16^ Recently, surface polarization has been proposed as a crucial trigger for hydride transfer on transition metals.^17^ Specifically, it was observed that more negative applied potentials increase the electrostatic potential drop at the metal/electrolyte interface, boosting the hydride transfer to acceptor molecules in the electrolyte.

Other than the nature of the catalyst, the electrolyte composition, especially the cations, has been shown to have a strong impact on the CO_2_RR activity and selectivity.^18−22^ On copper, silver, and gold electrodes,^22^ it was observed that the reduction of CO_2_ to CO does not occur in the absence of (alkali) cations in the electrolyte. This was ascribed to the stabilizing interaction between the cation and the first CO_2_ reduction intermediate (negatively charged surface-adsorbed CO_2_, denoted *CO_2_^δ−^), although other effects of alkali cations have also been suggested (regulation of local pH and/or the local double layer electric field).^22^ A HCOO^–^-mediated pathway to CO has been proposed to occur on Ag(111) via outer-sphere cation activation of CO_2_.^23^ These observations make it relevant to investigate the cation effect on HCOOH formation, for which the Pd_ML_Pt(111) electrode is an ideal model system.

In this work, we present CO_2_RR performance on a Pd_ML_Pt(111) electrode in a pH 3 electrolyte with various cation concentrations, focusing on selectivity differences between HCOOH and CO formation as a function of potential. We show that the onset potential for both HCOOH and CO formation shifts to a more positive potential with the increase of the cation concentration. Remarkably, there is a differential effect in the reaction paths, the cation effect being stronger on HCOOH formation than that on CO formation. Based on complementary density functional theory simulations, we present a mechanism to explain why HCOOH generation is highly sensitive to the presence of cations in the electrolyte under realistic conditions, i.e., high *H and *CO coverages. Our work underscores the crucial importance of cations and the overall microenvironment design in catalytic CO_2_RR.

## Experimental Section

### Chemicals and Cell Preparation

Ultrapure water (resistivity > 18.2 MΩ·cm, Millipore Milli-Q) was used for all experiments in this work. Prior to each experiment, all cell compartments were cleaned by storing them in a potassium permanganate solution (1 g L^–1^ KMnO_4_ (Fluka, ACS reagent) in 0.5 M H_2_SO_4_ (Fluka, ACS reagent)) overnight. The solution was subsequently drained, and the cell compartments were rinsed with a dilute piranha solution (1:3 v/v of H_2_O_2_ (Merck, Emprove exp)/H_2_SO_4_) to remove residual KMnO_4_ and MnO*_x_*. Afterward, the cell compartments were cleaned by repetitively rinsing and boiling with Milli-Q water to remove all inorganic contaminants. Electrolytes were prepared from LiClO_4_ (Sigma-Aldrich, ≥99.99% trace metal basis), NaClO_4_ (Sigma-Aldrich, ≥99.99% trace metal basis), KClO_4_ (Sigma-Aldrich, ≥99.99% trace metal basis), NaClO_4_ (Sigma-Aldrich, ≥99.99% trace metal basis), H_2_SO_4_ (Merck, Suprapur, 96%), PdSO_4_ (Sigma-Aldrich, 99.99% trace metal basis), and HClO_4_ (Sigma-Aldrich, Ultrapure, 70%). In this work, a Pt wire (0.5 mm diameter, MaTecK, 99.9%) was used as the counter electrode, a reversible hydrogen electrode (RHE) was used as the reference electrode, and all of the potentials were corrected for ohmic drop and controlled with an Autolab PGSTAT302N potentiostat.

### Preparation of Pd Monolayers on a Pt(111) Single Crystal

The characterization and preparation of the electrodes were carried out in the hanging meniscus configuration in standard three-electrode glass cells. Prior to each experiment, argon (Linde, 5.0) was purged through the electrolyte for 30 min to remove air from the solution. A Pt(111) single crystal (area = 0.08 cm^2^) was prepared according to the Clavilier method^24^ and characterized with cyclic voltammetry in 0.1 M HClO_4_. The cyclic voltammogram of Pt(111) in 0.1 M HClO_4_ measured at 50 mV s^–1^ is shown in Figure S1a. Afterward, the Pd monolayer was deposited on the Pt(111) single crystal with the method previously reported by our group.^16,25^ Briefly, the prepared Pt(111) single crystal was transferred and immersed into a Pd^2+^ containing electrolyte at +0.85 V versus RHE and then cycled between +0.07 and +0.85 V_RHE_ until a full monolayer of Pd was formed on Pt(111). The corresponding Pd formation process on Pt(111) is shown in Figure S1b, with the growth of a sharp peak at 0.23 V_RHE_ representing the accumulation of Pd on the Pt(111) surface and a decreasing peak at 0.5 V_RHE_, which is the characteristic spike of Pt(111) in 0.1 M H_2_SO_4_.^25^ With the accumulation of Pd on Pt(111), the peak at 0.5 V_RHE_ eventually disappears, which suggests a full monolayer on the Pt(111) surface. After Pd monolayer deposition, the Pd_ML_Pt(111) electrode was taken from the cell and rinsed with Milli-Q water thoroughly. Finally, the freshly prepared Pd_ML_Pt(111) was characterized in 0.1 M HClO_4_ and used as a working electrode in the subsequent experiments.

### Electrochemical Measurements

All electrochemical experiments were carried out in a standard three-electrode electrochemical cell. The CO_2_RR experiment in the absence of metal cations was performed in 1 mM HClO_4_ (pH 3). pH = 3 was chosen, as it is the highest pH where one still has reasonable electrolyte conductivity in the absence of (alkali) cations while having a low proton reduction current. In the case of electrolytes with different cation concentrations, calculated amounts of salt were added to a 1 mM HClO_4_ solution. Prior to each experiment, CO_2_ (Linde, 4.5) was purged through the electrolyte for at least 30 min to obtain a CO_2_-saturated electrolyte. Cyclic voltammetry measurements at 10 or 50 mV s^–1^ were first taken in pH 3 working electrolytes. Afterward, linear sweep voltammetry measurements were performed from 0.1 V_RHE_ to the required negative potentials at 1 mV s^–1^, followed immediately by CO oxidation stripping experiments taken at 10 mV s^–1^. The scan rate for CO oxidation stripping experiments was chosen to oxidize all CO adsorbed on the Pd surface in one scan. Due to the strong CO adsorption on the Pd surface,^25^ CO generated during CO_2_RR remains on the Pd surface until it is oxidized to CO_2_ during the CO oxidation stripping experiments. With a known surface area of the electrode (the same surface area as that of the Pt(111) single crystal), the CO surface coverage generated during CO_2_RR was then estimated from the CO oxidation charge obtained from the stripping experiments.^16,26^ In the case of linear sweep voltammetry recorded in an argon-purged electrolyte, argon was first purged through the electrolyte for at least 30 min to obtain an air-free electrolyte, and then linear sweep voltammetry experiments were performed from 0.1 V_RHE_ to negative potential at a scan rate of 10 mV s^–1^.

### Online High-Performance Liquid Chromatography (HPLC)

All of the online HPLC experiments were carried out in a H-type electrochemical cell equipped with three electrodes. The cell compartments were separated by a Nafion 117 membrane. Prior to the online HPLC experiments, CO_2_ (Linde, 4.5) was purged through the cell for at least 30 min to saturate the electrolyte. While linear sweep voltammetry was performed from +0.1 V_RHE_ to the required negative potentials at 1 mV s^–1^, liquid samples were simultaneously collected with an open tip positioned close to our working electrode at a collection rate of 60 μL min^–1^.^27^ Therefore, each sample contained products averaged over a potential range of 60 mV. Afterward, the collected samples were analyzed by HPLC equipped with an Aminex HPX-87H column (BioRad) and a RID detector (Shimadzu). Notably, the obtained HCOOH concentration in this work is a measure of the HCOO^–^ concentration near the working electrode (which will be higher than bulk HCOO^–^ concentration) and therefore is not suitable for quantitative Faradaic efficiency determination. Chronoamperometry experiments at −1.2 V_RHE_ were performed to check HCOOH formation in a pH 3 electrolyte in the absence of metal cations. The online HPLC tip was positioned close to the working electrode. After 10 min of chronoamperometry, a liquid sample was taken and further analyzed with HPLC. A higher local HCOOH concentration (if there is any formed during CO_2_RR) with chronoamperometry at fixed potentials is expected and detected with HPLC.

### Density Functional Theory Details

We performed the density functional theory (DFT) simulations through the Vienna Ab initio Simulation Package (VASP).^28,29^ We employed the PBE density functional^30^ including dispersion through the DFT-D2 method,^31,32^ with our reparametrized C_6_ coefficients.^33^ Inner electrons were reproduced by PAW pseudopotentials,^34,35^ and the monoelectronic states for the valence electrons were expanded as plane waves with a kinetic energy cutoff of 450 eV. We modeled the experimental system in agreement with our recent works.^22,36^ The surface model for the cation-free case consisted of a (3 × 3) Pd_ML_Pt(111) supercell, including 1 Pd monolayer on top of 4 Pt layers, where the two bottom layers were kept fixed to the bulk distance (see Figure 4a,b). To assess the role of alkali cations, we introduced in the simulation cell a solvated K^+^ (with 5 H_2_O in its coordination shell), fixing their *z*-coordinate to a distance of 4 Å (near-cation) and 9 Å (far-cation) from the surface layer. We considered two systems with different cation–surface distances to decouple short- and long-range cation interactions. Besides, to assess the case of charged simulations cells,^37^ we considered for both the near- and far-cation cases a cell with 1 excess electron (K^+^ without OH^–^) and a neutral cell (K^+^ with OH^–^). For the second system, K^+^ was neutralized via a OH^–^ formed by removing a hydrogen from one of the H_2_O molecules.^22^ The vacuum extended for at least 10 Å beyond the cation’s solvation shell (or the Pd surface layer, for the cation-free case). Since the solvation layer with cations, water molecules, and adsorbates was placed only on one side of the slab, an additional dipole correction was applied to remove spurious contributions arising from the asymmetric slab model.^38^

### Effect of Applied Electric Potential on Formation Energies

The thermodynamics of CO_2_ activation was assessed by computing the formation energy of this intermediate (eqs 1 and 2). CO_2_ (1/9 ML) was let to adsorb on the surface. For the system with a 4 Å distance between K^+^ and the surface, the adsorbate was initially placed in proximity of the alkali cation (*d*_K^+^–O(CO_2_)_ ∼ 2.80 Å)^39^ to enable short-range cation effects. Formation energies were calculated taking as energy references CO_2_(g), H_2_O(g), and surface + solvation layer, i.e., Pd_ML_Pt(111) 3 × 3 + K^+^(5H_2_O). To account for variations in the metal work function and consequent changes of electric potential along the reaction path, we first estimated DFT formation energies at the electrostatic potential Φ corresponding to the work function *W* of the environment. The electrostatic potential Φ was calculated by normalizing the work function by the unit of elementary charge *e*^–^ (–1*e*^–^).^40^*E* and Φ indicate, respectively, DFT energies and electrostatic potential for surface + solvation + adsorbate (ads) and surface + solvation alone (surf), as reported in Table S1, while *q* represents the surface charge density for the initial state (surface + solvation) and final state (surface + solvation + adsorbate), see Table S2. Next, we computed all energies at the work function corresponding to an applied electric potential of −0.40 V versus RHE (i.e., −0.58 V vs SHE at pH = 3) through eq 2.^41^ Given that the work function for the standard hydrogen electrode is 4.4 eV,^40^ at an applied potential of −0.4 V versus RHE, the expected metal work function is 3.82 eV. Δ*q* represents the change in surface electronic charge along the reaction step, and it is given in unit of elementary charge *e*^–^ (−1*e*^–^).
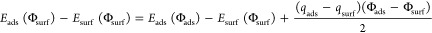
1
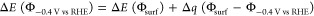
2

## Results

### Cyclic Voltammetry of PdmLPt(111)

**Figure 1:**
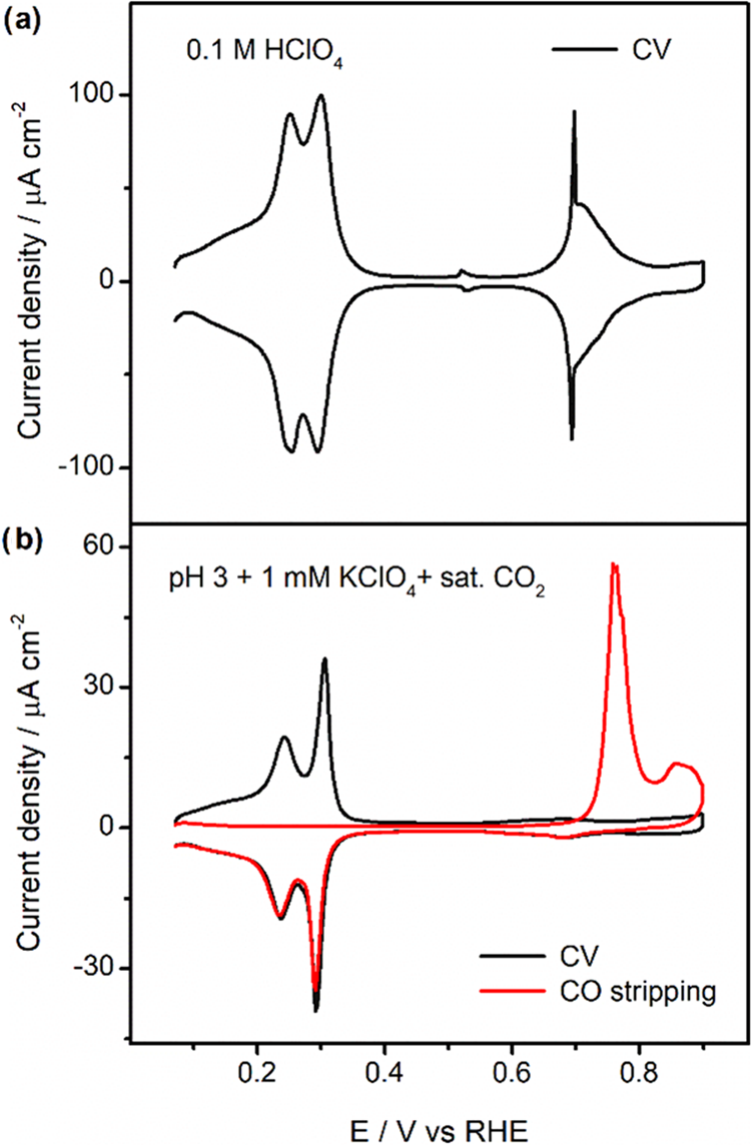
Cyclic voltammetry of Pd_ML_Pt(111). (a) Cyclic voltammogram of the Pd_ML_Pt(111) electrode in 0.1 M HClO_4_. Scan rate: 50 mV s^–1^. (b) Blank cyclic voltammograms (black) and CO stripping voltammogram (red) of Pd_ML_Pt(111) in a CO_2_-saturated pH 3 electrolyte in the presence of 1 mM KClO_4_. Scan rate: 10 mV s^–1^.

Figure 1a shows the blank cyclic voltammogram of Pd_ML_Pt(111) in 0.1 M HClO_4_. The blank curve, measured at 50 mV s^–1^, shows the characteristic regions of the Pd_ML_Pt(111) electrode: a region between +0.05 and +0.35 V_RHE_ with peaks at +0.21 and +0.31 V_RHE_, corresponding to the replacement of adsorbed *H by adsorbed *OH and the replacement of adsorbed *OH by adsorbed *ClO_4_^–^, respectively; a low-current region between +0.35 and +0.65 V_RHE_ in which the surface is covered with adsorbed perchlorate anions (which appear to undergo a structural transition at ca. +0.52 V_RHE_); and a region between +0.65 and +0.90 V_RHE_ with a sharp peak at +0.69 V_RHE_, which is ascribed to the replacement of adsorbed *ClO_4_^–^ by a higher coverage of *OH adsorption or *O adsorption.^16,25^ The blank voltammetry in Figure 1a confirms the deposition of a single Pd monolayer on the Pt(111) single crystal as well as the cleanliness of the electrolyte, thereby ensuring the robustness and reproducibility of the experiments in this work.

There are small changes and shifts in these peaks for a pH 3 electrolyte in the absence and presence of cations, which have been described in detail in previous work.^25^Figure 1b shows the blank cyclic voltammogram of Pd_ML_Pt(111) in a pH 3 electrolyte with 1 mM KClO_4_ in the presence of CO_2_. CO_2_ leads to the presence and adsorption of bicarbonate (HCO_3_^–^) on Pd_ML_Pt(111). As shown in the black curve in Figure 1b, the peak at +0.31 V_RHE_ increases in sharpness (compared with the peak at +0.21 V_RHE_), while the stronger bicarbonate adsorption than perchlorate adsorption lowers the current density in the potential window from +0.35 to +0.9 V_RHE_, i.e., it suppresses *OH and/or *O adsorption.^25^ Importantly, below +0.1 V_RHE_, the Pd_ML_Pt(111) electrode is fully covered with adsorbed hydrogen.

Voltammetry is also used to estimate the amount of adsorbed carbon monoxide (*CO) formed on the Pd_ML_Pt(111) electrode during CO_2_RR experiments. To this end, we use CO stripping voltammetry. The red curve in Figure 1b shows the oxidative stripping voltammogram of a full saturation coverage of *CO obtained from CO_2_RR on the Pd_ML_Pt(111) electrode, which corresponds to ca. 0.75 ML in terms of available Pd surface atoms. *CO remains on the surface of the Pd_ML_Pt(111) electrode after CO_2_RR, which blocks the electrode surface and results in a low current observed in the region between +0.05 and +0.35 V_RHE_ at the beginning of the oxidative stripping voltammogram. With more positive potential, CO oxidation peaks between 0.65 and 0.90 V_RHE_ are observed. Upon oxidation of adsorbed *CO, the typical CV features in a pH 3 electrolyte are again observed in the negative going scan. This means that the Pd_ML_Pt(111) electrode should still be intact and can be used again for a new experiment.

### Cation Effect on HCOOH and CO Formation during CO_2_RR

**Figure 2:**
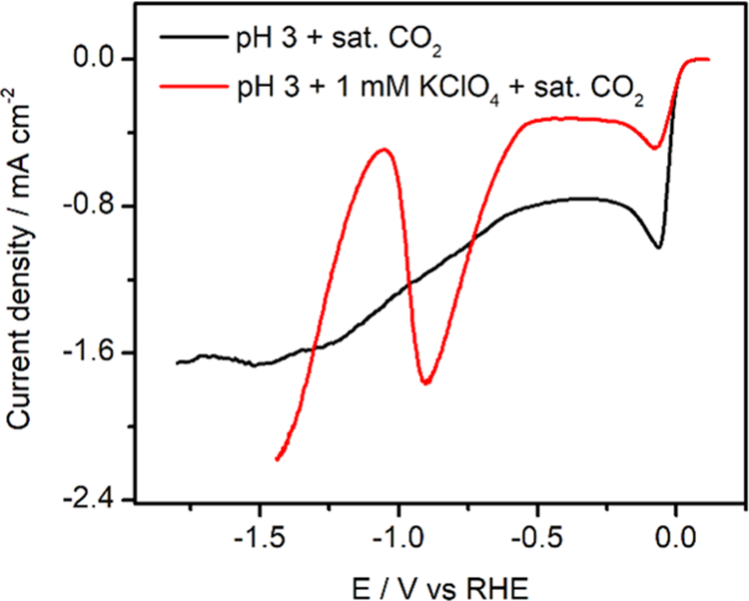
Linear sweep voltammetry of Pd_ML_Pt(111) in CO_2_-saturated pH 3 electrolytes in the absence (black) and presence of 1 mM KClO_4_ (red). Scan rate: 10 mV s^–1^.

After surface characterization, we studied the cation concentration effect on formic acid and CO formation during CO_2_RR on Pd_ML_Pt(111) in pH 3 electrolytes. The CO_2_RR experiments were carried out by linear sweep voltammetry, scanning from +0.07 V_RHE_ to different negative vertex potentials at 1 mV s^–1^. HCOOH and CO production during CO_2_RR was obtained from online HPLC and CO stripping voltammetry, respectively, as introduced in the Experimental Section and the previous section. Figure 2 shows the corresponding linear sweep voltammograms obtained at a higher scan rate (10 mV s^–1^). The meaning of the effect of 1 mM KClO_4_ on the voltammetry is explained in the following sections. All CO stripping voltammograms obtained in this work are shown in Figures S3–S7 in the Supporting Information.

**Figure 3:**
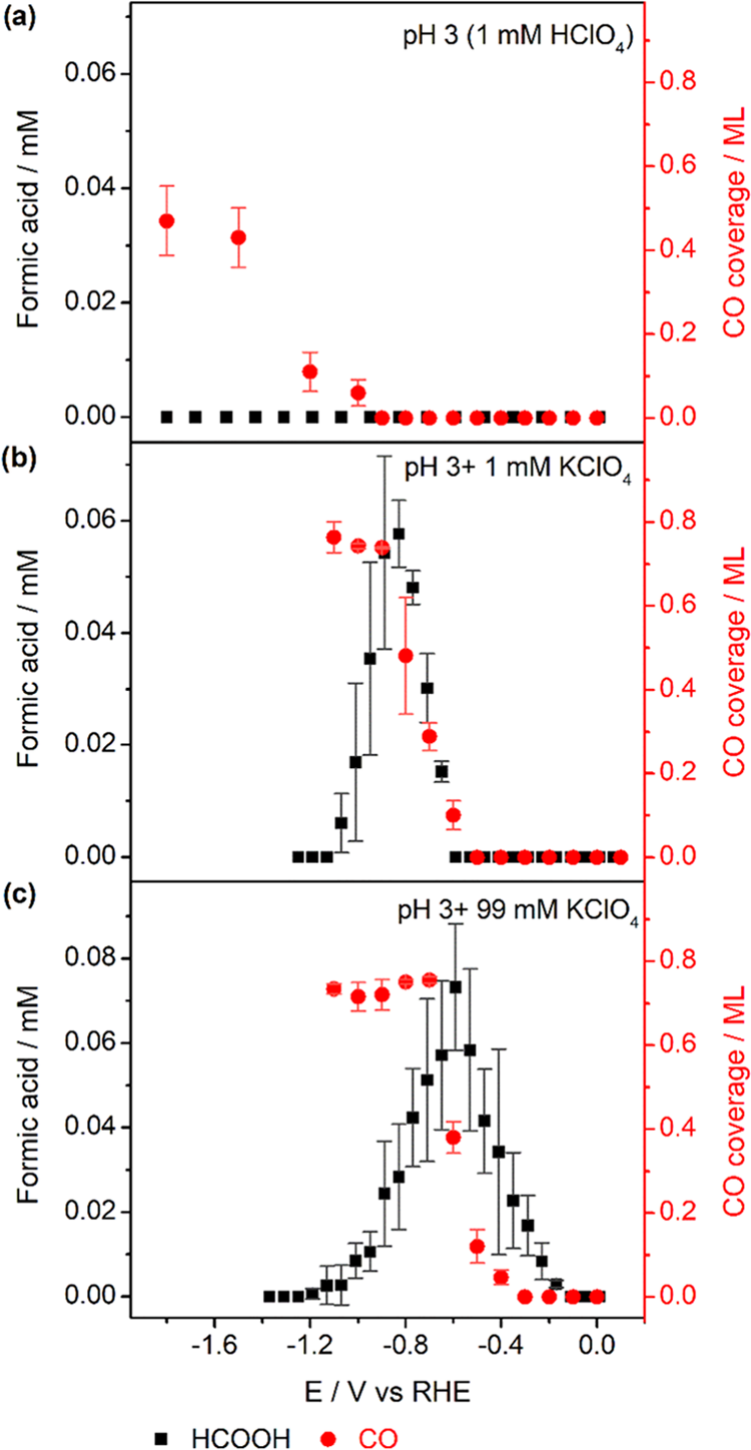
Cation concentration effect on CO_2_RR on Pd_ML_Pt(111). CO coverage and HCOOH formation during CO_2_RR obtained in CO_2_-saturated pH 3 electrolytes (a) in the absence of metal cations and in the presence of (b) 1 mM KClO_4_ and (c) 99 mM KClO_4_. Error bars are standard deviations based on three independent measurements.

Figure 3 shows the obtained CO coverage and HCOOH production during CO_2_RR on Pd_ML_Pt(111) as a function of potential in a pH 3 electrolyte in the presence of 0, 1, and 99 mM KClO_4_ separately (additional data for pH 3 with 5 and 10 mM KClO_4_ are shown in Figures S5 and S6, and a comparison is provided in Figure S8). Figure 3a shows CO and HCOOH production in the absence of metal cations. CO is observed on Pd_ML_Pt(111) in the absence of metal cations, albeit at a considerably negative onset potential of −1.0 V_RHE_. The obtained maximum CO coverage under these conditions is ca. 0.4 ML at −1.8 V_RHE_, which is much lower than the saturation CO coverage reported before.^16^ Remarkably, no HCOOH production was observed in the absence of metal cations. To verify this result, 10 min of chronoamperometry was also carried out at −1.2 V_RHE_ with online HPLC, and indeed no HCOOH was detected (see Figure S3b). Although reporting Faradaic efficiencies (FEs) is not the goal of this work, note that the amount of HCOOH formed is below the limit of detection for HPLC, whereas that of CO (determined by stripping voltammetry) is not, at more negative potentials. However, the CO that we detect is adsorbed CO, not dissolved CO. We have no (voltammetric) evidence for the formation of dissolved CO under the conditions of our experiment.

Figure 3b,c shows the same experiments with different concentrations of metal cations in the electrolyte. Figure 3b shows CO and HCOOH formation during CO_2_RR in the electrolyte with the presence of 1 mM KClO_4_. *CO is produced with an onset potential of −0.6 V_RHE_, whereas the formation of HCOOH was observed with a slightly more negative onset potential of −0.65 V_RHE_. With the increasingly negative going potential, *CO coverage and HCOOH formation keep increasing until a high *CO coverage (ca. 0.74 ML) is reached at −0.9 V. From −0.9 V_RHE_ and more negative potentials, the *CO coverage remains constant, while HCOOH formation drops due to the high coverage of *CO on the surface. Compared with CO_2_RR in a pure pH 3 electrolyte, it is clear that the onset potential of CO_2_RR is less negative in the presence of 1 mM KClO_4_. With the increase of the cation concentration (99 mM KClO_4_, Figure 3c), CO formation starts at −0.4 V_RHE_, whereas HCOOH is produced at −0.2 V_RHE_. This result shows that cations have a stronger effect on HCOOH production than that on *CO formation, yielding a lower overpotential for HCOOH in the presence of a high cation concentration. This result is consistent with HCOOH as the major CO_2_RR product (with minor CO poisoning) at low overpotentials, as shown in previous work on nanoparticulate Pd. Note that on nanoparticulate Pd, the current due to H intercalation should be minimal, at least during long-term electrolysis experiments.^3^

Knowing the effect of cations of CO_2_RR on Pd_ML_Pt(111), we can now understand the voltammograms in Figure 2. The reduction wave between 0 and −0.5 V_RHE_ is due to the (diffusion-limited) reduction of protons (their concentration being ca. 1 mM at pH = 3). The current density is higher in the absence of cations (black curve), as under these conditions, protons are also transported to the surface by migration, in addition to diffusion.^42,43^ In the presence of cations (red curve), CO_2_ reduction starts after −0.5 V_RHE_. The corresponding peak is completely absent when cations are lacking. The peak shape is caused by the fact that at potentials more negative than −0.9 V_RHE_, adsorbed CO is formed, and the production of HCOOH is inhibited. To confirm the origin of the peak at −0.8 V_RHE_, linear sweep voltammetry was carried out under the same experimental conditions in the argon-purged pH 3 electrolyte. The corresponding linear sweep voltammogram is shown in Figure S2. No peak was observed in the pH 3 electrolyte with the presence of 1 mM KClO_4_, confirming that the peak at −0.8 V_RHE_ is due to CO_2_ reduction.

Interestingly, at potentials more negative than −1.1 V_RHE_, the solution with cations clearly shows the reduction of water. There is no such clear water reduction current in the absence of cations. One could interpret this as an important promoting role of cations of water reduction (an effect which is known),^44^ but one should also be aware that in the absence of supporting electrolytes, there are no ions available to carry such a reduction current. Therefore, we cannot claim that water reduction does not occur without cations, only that cations promote water reduction.

**Figure 4:**
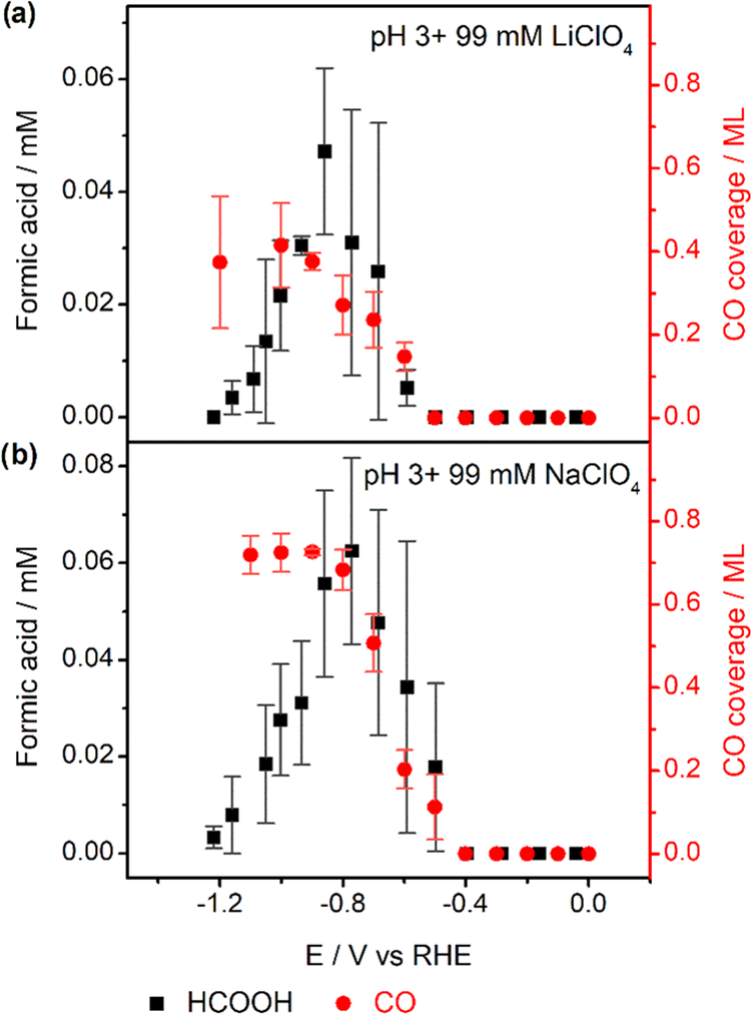
Cation identity effect on Pd_ML_Pt(111). CO coverage and HCOOH formation during CO_2_RR obtained in CO_2_-saturated pH 3 electrolytes containing 99 mM (a) LiClO_4_ and (b) NaClO_4_. Error bars are standard deviations based on three independent measurements.

Furthermore, we also studied the effect of cation identity on CO and HCOOH formation during CO_2_RR on Pd_ML_Pt(111). Figure 4 shows the obtained CO coverage and HCOOH production during CO_2_RR as a function of potential in pH 3 electrolytes in the presence of 99 mM LiClO_4_ and NaClO_4_, respectively. We observe that the CO coverage obtained from the pH 3 electrolyte containing 99 mM LiClO_4_ is ca. 0.6 ML, which is less than the coverage obtained in the pH 3 electrolyte containing 99 mM NaClO_4_ or KClO_4_. Moreover, we observe a correlation between CO_2_RR activity and the cation identity. The activity for CO and HCOOH formation on Pd_ML_Pt(111) increases in the order Li^+^ < Na^+^ < K^+^, following the ability of these species to accumulate near the surface.^22,36^ These results confirm the specific importance of the nature of the metal cations and that, in general, a strongly hydrated cation such as Li^+^ has a lower promoting effect on CO_2_ activation than more weakly hydrated cations.

### Computational Model

To gain additional insights into the mechanism underlying the observed cation effects on HCOOH and CO formation during CO_2_RR, we carried out complementary density functional theory (DFT) simulations. According to the state of the art, the mechanisms for the formation of formic acid and CO from CO_2_RR encompass several elementary steps. For CO_2_ reduction to formic acid on Pd, it is now agreed that the most relevant step is the adsorption of hydrogen as a hydride (*H^δ−^),^3,16^ which performs a nucleophilic attack on the positively charged carbon of nearby CO_2_ in solution. Depending on the bulk solution pH, the generated formate becomes protonated to formic acid. Instead, the route toward CO requires the adsorption of CO_2_ via the C atom, with subsequently one of the terminal O atoms becoming hydrogenated until water splits off, leaving adsorbed *CO on the catalyst surface.

**Figure 5:**
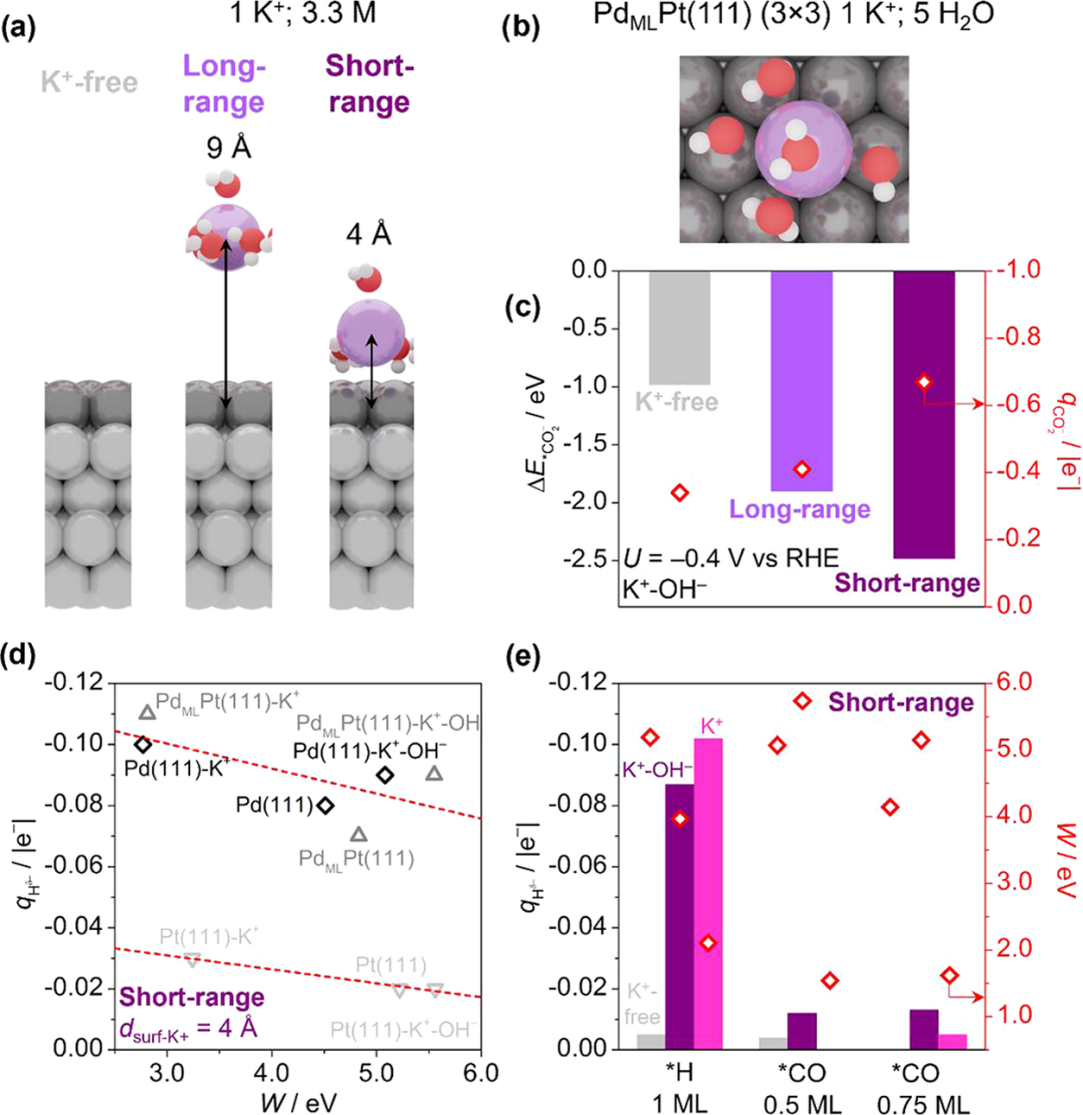
Cation promotional effect on CO_2_ adsorption and formation of a surface hydride (*H^δ−^). (a) Side and (b) top views of the Pd_ML_Pt(111) (3 × 3)-1 K^+^ (5 H_2_O) models. (c) CO_2_^–^ formation energy at *U* = −0.4 V versus RHE (left *y*-axis) and excess charge on the CO_2_ unit (right *y*-axis) for a neutral Pd_ML_Pt(111) (3 × 3)-1 K^+^ (5 H_2_O) supercell (K^+^–OH^–^ couple). (d) Correlation between H^δ−^ Bader charges and work function (*W*) of Pd(111) (3 × 3), Pd_ML_Pt(111) (3 × 3), and Pt(111) (3 × 3), respectively black, dark, and light gray data points. For both charged (K^+^) and neutral supercells (K^+^–OH^–^), the cation-surface distance was 4 Å. (e) H^δ−^ Bader charges (left *y*-axis) and metal work function (red data points, right *y*-axis) for Pd_ML_Pt(111) (3 × 3) at various *H and *CO coverages. Gray, purple, and magenta columns indicate, respectively. K^+^-free, neutral (K^+^–OH^–^), and charged (K^+^) supercells. When present, the cation was placed 4 Å far away from the surface.

To model these key reaction steps, we carried out DFT simulations with the PBE-D2 functional.^30−33^ To simulate the experimental surface without cation in the electrolyte, we chose a (3 × 3) Pd_ML_Pt(111) supercell, composed of 1 Pd monolayer on top of 4 Pt layers, of which the two lower layers were kept fixed to resemble the bulk. Further, to assess the role of metal cations, we included 1 K^+^ with 5 coordinated H_2_O molecules within its solvation shell in the simulation cell.^36^ Since the volume of the solvation layer accounts for 0.50 nm^3^ (7 Å thickness), the surface cation concentration was equivalent to 3.3 M. K^+^ was inserted at two fixed distances either 4 or 9 Å from the Pd_ML_Pt(111) surface (Figure 5a,b) to decouple short- and long-range cation effects on reaction intermediates. To avoid artificial interactions between periodic images, the vacuum thickness extended for 10 Å beyond the Pd_ML_Pt(111) surface layer for the cation-free case and beyond the outermost water molecule if the cation was present.^45^ Since we recently highlighted the importance of charged simulation cells to model cation effects,^37^ we considered here both a cell with 1 excess electron (K^+^ without OH^–^) and a neutral cell (K^+^ with OH^–^). In this second case, we neutralized K^+^ via a OH^–^ formed by removing a hydrogen from one of the H_2_O molecules.^22^ Since excess electrons in the simulation cell led to significant variations in the metal work function *W* and consequently in the electrostatic potential Φ along the simulation cell (Figures S9 and S10), when assessing the thermodynamics of CO_2_ activation (Figure 5c), we opted for neutral supercells (K^+^–OH^–^ couple) to keep *W* constant (as shown in Table S1).

### Mechanism of the Cation Effect on CO_2_ Activation

CO_2_ activation is often assumed as the rate-determining step for CO_2_RR on transition metals.^22^ To assess the potential role of cations on CO_2_ reduction to CO, we estimated the energy required for activating CO_2_ on Pd_ML_Pt(111) (3 × 3) for the cation-free case (see eq 3) and in the presence of cations at 4 and 9 Å far away from the surface (eq 4). We corrected the DFT energy for a potential-dependent term calculated at *U* = −0.4 V versus RHE (pH = 3), the observed onset potential for CO_2_ reduction to CO (Figure 3c). Such a potential-dependent term was obtained by tuning the metal work function in line with the approach suggested by Chan and Nørskov (refs (40, 41), as shown in Effect of Applied Electric Potential on Formation Energies, eqs 1 and 2, and Tables S1 and S2). In eq 5, Δ*q* represents the change in electronic surface charge for Pd_ML_Pt(111) (3 × 3) between the final (surface + *CO_2_^–^) and initial (surface + CO_2_) states; thus, it is equal to −1*e*^–^.
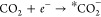
3
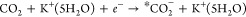
4
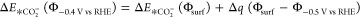
5

In the presence of a proximal metal cation (*d*_K^+^-surf_ = 4 Å), the K^+^ coordinates to the free oxygen of the CO_2_ molecule, leading to strong CO_2_ binding (Δ*E* = −2.5 eV, as reported in Figure 5c). Instead, for a cation-surface distance of 9 Å, we observe a weaker *CO_2_ adsorption energy of around −1.9 eV. In agreement with experimental results (see Figure 3a), CO_2_ activation occurs also for the cation-free case, being exothermic by around −1.0 eV at −0.4 V versus RHE. Additionally, our results show that metal cations enhance the electron transfer from Pd_ML_Pt(111) to adsorbed CO_2_ (see the right *y*-axis in Figure 5c). In fact, the CO_2_ Bader charge (*q*_CO_2__) is −0.67 |*e*^–^| for *d*_K^+^-surf_ = 4 Å, while it decreases to −0.41 |*e*^–^| if the cation is far from the surface (*d*_K^+^-surf_ = 9 Å). *q*_CO_2__further reduces to −0.34 |*e*^–^| in the absence of metal cations in the simulation cell. Cation-promoted electron transfer to CO_2_ was confirmed as well on Pd(111) (3 × 3) and Pt(111) (3 × 3), see Figure S11.

### Mechanism of the Cation Effect on Hydride Formation

Moving on to HCOOH selectivity, we assess the effect of cations on the formation of the adsorbed hydride (*H^δ−^). As shown in Figure 5d, the Bader charge of the adsorbed hydride (*H^δ−^) correlates with the work function of Pd(111), Pd_ML_Pt(111), and Pt(111), in line with previous findings in refs (3, 16) In turn, the metal work function decreases in the presence of neighboring K^+^ due to increased cation-induced polarization of the surface electronic density, favoring the formation of surface hydrides. Such a phenomenon may also be expressed in terms of the metal d-band center. For materials with a low d-band center, such as Pt(111) (ε_d_ ∼ −2.0 eV), the limited electronic density available at the surface prevents the effective formation of a hydride (δ^–^ ∼ −0.02 |*e*^–^|). Instead, on metals characterized by higher values of the d-band center (*ε*_d_ ∼ −1.5 eV, low work function), such as Pd(111) and Pd_ML_Pt(111), more electronic density is available at the surface, enabling the formation of surface hydrides (δ^–^ ∼ −0.10 |*e*^–^|). A previous experimental study showed a strong correlation between Pd content and HCOOH/HCOO^–^ selectivity on Pt–Pd nanoparticles and aerogels, while pure Pt was reported not able to reduce CO_2_ to HCOOH/HCOO^–^.^16,27,46^ We here confirm that CO_2_RR to HCOOH requires hydride species on PdPt catalysts, which occur only for suitable surface charging, i.e., appropriate filling of *d*-band states or induced by the neighboring cation. In fact, we observe that *H^δ−^ Bader charges correlate with the K^+^-induced electric field (proportional to *q*^K^+^^/*r*_K^+^-H_^2^), as shown in Figure S12 and Table S3. Higher intensities of the cation-induced electric field lead to increased polarization at the surface,^17^ which enhances the hydridic character of *H.

Figure 5d suggests that surface hydrides should also be formed in the absence of cations, which is at odds with experimental results on HCOOH selectivity for Pd_ML_Pt(111) (Figure 3a). Thus, we upgraded our model to more realistic conditions, i.e., high *H and *CO coverages, retrieving input geometries from a previous theoretical study.^47^ Remarkably, hydrides are preserved at 1 ML *H coverage only in the presence of neighboring cations, while δ^–^ > −0.01 |*e*^–^| in the cation-free case (Figure 5e). High CO coverages (0.5 and 0.75 ML) hinder the formation of surface hydrides both in the absence and presence of alkali cations. Both insights can be rationalized by the upshift of the Pd_ML_Pt(111) work function at high adsorbate coverages, partially balanced by the downshift in the presence of the neighboring cation at high *H coverage (Figure 5e). Other factors may also contribute to the selective reduction of CO_2_ to HCOOH on Pd_ML_Pt(111) at mild negative potentials. Considering the case of outer-sphere CO_2_ activation to HCOO^–^ by alkali cations, proposed by Pidko et al.^23^ on Ag(111), such coupling is exothermic for both neutral (K^+^–OH^–^) and charged (K^+^) cells (see Figure S13) on Pd_ML_Pt(111) (3 × 3). Instead, for the cation-free case, such process is endothermic by 0.2 eV. Nevertheless, in the case of adsorbed hydride transfer to physisorbed CO_2_ at −0.4 V versus RHE, the cation contribution appears less critical. In fact, such a step is almost thermoneutral (∼0.1 eV) both in the presence and absence of neighboring cations (Figure S13).

## Discussion

Our results show the crucial role of metal cations on HCOOH and CO formation during CO_2_RR on Pd_ML_Pt(111). Without metal cations, only limited CO_2_ reduction to CO takes place.^22^ This result is slightly different from the previous work reported by our group for CO_2_ activation to CO on Ag, Au, and Cu electrodes, for which cations are a necessary reaction partner. This difference must be due to the fact that palladium binds CO more strongly, and therefore, the activation barrier for CO formation is lower, such that even in the absence of cations, CO formation can take place. The activity toward HCOOH and CO improves with the increase of cation concentration, leading to the decreased onset potential of both products. Remarkably, Pd_ML_Pt(111) produces no HCOOH in the absence of cations, while it is still somewhat active for *CO formation. In the presence of a high cation concentration, the selectivity pattern reverses and HCOOH formation happens at a lower overpotential, suggesting a stronger cation effect on HCOOH formation than that on CO formation on Pd_ML_Pt(111). In previous work, we have rationalized the role of *H^δ−^ on HCOO^–^/HCOOH formation on Pd surfaces and the dependence of hydridic character on the metal work function.^16^ Hydrides are instrumental in reducing CO_2_ to HCOO^–^/HCOOH on Pt-Pd catalysts through a nucleophilic attack on nearby CO_2_.

The insights from our DFT simulations support the mechanism reported for CO versus HCOO^–^/HCOOH competition on Pd_ML_Pt(111),^16^ complementing our previous observations on other transition metals (Ag, Au, Cu).^22^ Neighboring cations (*d*_K^+^-surf_ = 4 Å) enhance CO_2_ activation by more than 1 eV at *U* = −0.4 V_RHE_ versus the cation-free case, further facilitating the electron transfer to the CO_2_ unit (CO_2_ Bader charge of −0.67 |*e*^–^|). Regarding formic acid/formate selectivity, hydrides are effectively formed on materials with a low work function, such as Pd(111) and Pd_ML_Pt(111). These catalysts enable the transfer of surface electronic density to the adsorbate, while hydrides are not formed on catalysts with deep electronic states, such as Pt(111), which prevent the formic acid pathway. In case of a high *H coverage, the cation-induced local electric field counterbalances the upshift of the metal work function due to the adsorbates, which instead hinder the formation of hydrides for the cation-free case. Such evidence rationalizes one of the most striking experimental observations in this work, i.e., the complete absence of HCOOH formation in the absence of cations.

In addition to the *H coverage effect, cation-induced outer-shell CO_2_ activation and short-range stabilization of HCOO^–^ may also aid in favoring HCOOH formation.^23^ However, given the general agreement that HCOOH selectivity crucially depends on hydrides, we highlight here the stabilization of the hydridic character as the critical contribution of cations. We note that cations enhance the formation of surface hydrides and the hydride transfer to CO_2_ by inducing further polarization of the Pd–H bond, in line with the conclusions of ref (17). Finally, during CO_2_RR, the interfacial pH changes,^20,48^ which may impact interfacial reactivity. Previously reported work suggested a pH swing close to a Au electrode from bulk pH conditions to an interfacial pH of around 10–12 during CO_2_RR under highly reactive conditions.^20,48^ Cations have a small buffering capacity,^21^ with an impact on the interfacial pH, which can be measured.^48^ This effect may be one of the factors contributing to our experimental results. However, it is hard to imagine that this relatively small effect would explain the observations shown in Figure 3.

## Conclusions

We have studied the cation effect on HCOOH and CO formation during CO_2_RR with a well-defined Pt(111) electrode with an epitaxially grown Pd monolayer. The experiments show that cations have a stronger effect on HCOOH formation than that on *CO formation. There is no HCOOH formation in the absence of cations, though there is *CO formation at high overpotentials. In the presence of a high cation concentration, a lower overpotential for HCOOH formation is observed. Our DFT results help rationalize the experimental observations. Both CO and HCOOH formation pathways involve negatively charged intermediates, i.e., negatively charged adsorbed CO_2_ and adsorbed hydride, respectively, whose formation is favored by the interaction with cations. The generation of hydrides is favored by a low metal work function (that correlates with an appropriate position of the metal d-band). The cation can further downshift the metal work function by inducing increased surface electronic density and thus promoting hydrides or sustaining the formation of hydrides at high *H coverage. Consequently, CO_2_ reduction to formic acid takes place at mild applied potentials. Instead, high CO coverages prevent the formation of hydrides both in the presence and absence of cations, causing the quick decrease of formic acid selectivity at high applied potentials. As a final remark, since cation effects on CO_2_RR to HCOOH depend on both cation accumulation at the surface (well described by cation acidity) and the induced electronic density at the surface, further studies to optimize electrolytes toward formic acid selectivity should target cations (or mixtures) with a high induced electric field (i.e., *H^δ−^ favorable formation) and relatively low acidity, beyond the current employed standard cations (Figure S12 and Table S3).

Our study reveals once more that the search for optimized systems for CO_2_R should carefully tailor the design of both sides of the electrolyte/electrode interface, especially for HCOOH/HCOO^–^ formation.

## Data Availability

The data sets generated through DFT simulations and analyzed during the current study are available in the ioChem-BD database (ref (49)) at DOI: 10.19061/iochem-bd-1-273.^50^ Experimental data sets are available from the corresponding author upon reasonable request.

## Supporting Information Available

Additional cyclic voltammetry data; additional HPLC data; and additional computational data (PDF)
