# Supplementary material for: Influence of Cations on HCOOH and CO Formation during CO2 Reduction on a PdMLPt(111) Electrode
Source: J Am Chem Soc. 2023 Aug 31;145(36):19601–10. doi: 10.1021/jacs.3c03786 (PMC10510319; doi:10.1021/jacs.3c03786)
Supplement: Supplementary file 1 — ja3c03786_si_001.pdf [file ja3c03786_si_001.pdf]

Influence of Cations on HCOOH and CO Formation during CO_2 Reduction on a $Pd_{ML}Pt(111)$ Electrode

Chunmiao Ye[†, ‖], Federico Dattila[‡, §, ‖], Xiaoting Chen[†], Núria López[‡], Marc T.M. Koper[*, †]

[†] Leiden Institute of Chemistry, Leiden University, 2300 RA Leiden, The Netherlands

[‡] Institute of Chemical Research of Catalonia (ICIQ-CERCA), The Barcelona Institute of Science and Technology (BIST), 43007 Tarragona, Spain

[§] Present Address: Department of Applied Science and Technology (DISAT), Politecnico di Torino, Corso Duca degli Abruzzi 24, 10129 Turin, Italy.

[‖] C.Y. and F.D. contributed equally to this work.

Supporting Information

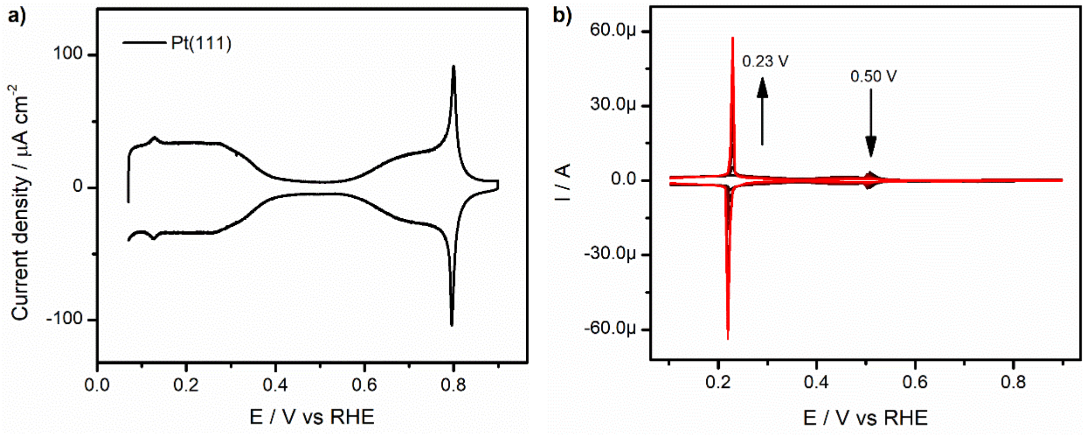

Figure S1 a. Cyclic voltammogram of Pt(111) in 0.1 M $HClO_4$. Scan rate: 50 mV s^{-1}. **b.** Cyclic voltammograms for Pt(111) in 0.1 M H_2SO_4 + 0.1 mM $PdSO_4$, recorded in successive stages during Pd deposition experiment. Scan rate: 50 mV s^{-1}. Arrows indicate the evolution with time.

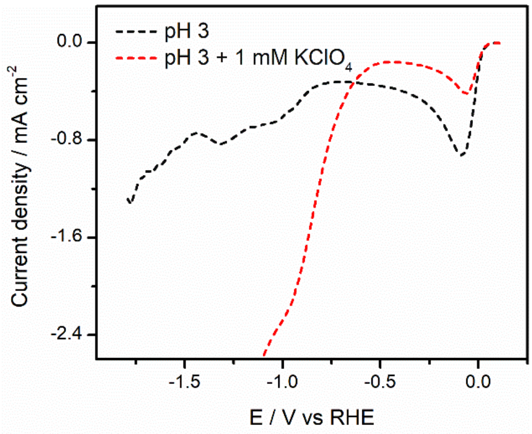

Figure S2. Linear Sweep Voltammetry of $Pd_{ML}Pt(111)$ in argon-purged pH 3 electrolytes in the absence (black) and presence of 1 mM $KClO_4$ (red). Scan rate: 10 mV/s.

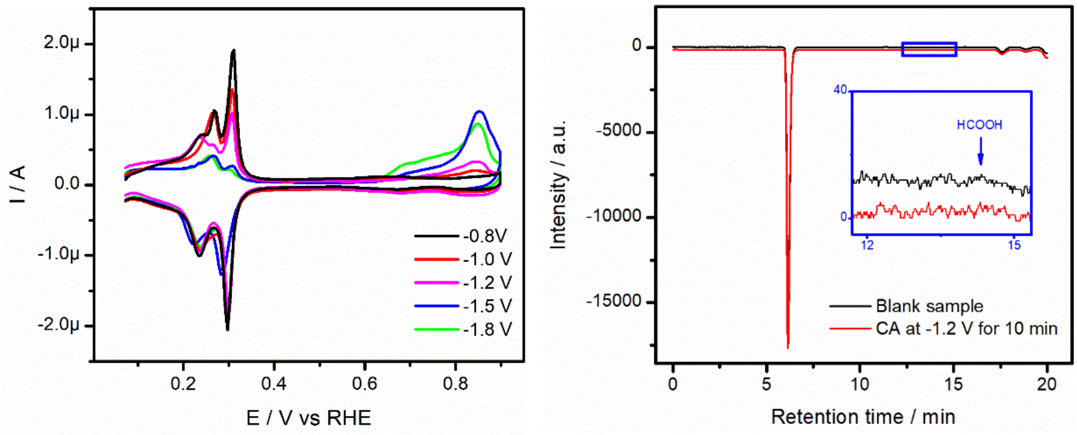

Figure S3 a. CO stripping voltammograms on $Pd_{ML}Pt(111)$ after CO_2 reduction at different vertex potentials in CO_2-saturated pH 3 electrolyte without metal cations, measured at 10 mV s^{-1}. **b.** HPLC data before and after chronoamperometry at –1.2 V_{RHE} for 10 min in CO_2-saturated pH 3 electrolyte without metal cations.

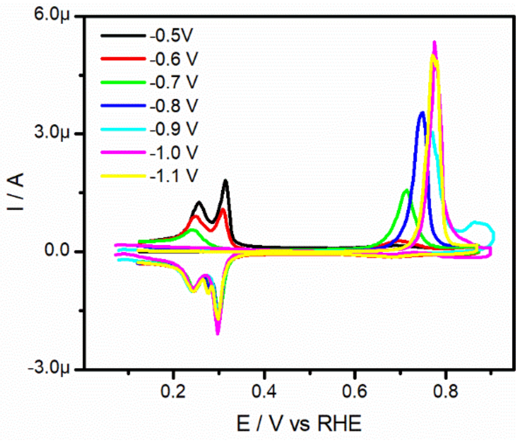

Figure S4. CO stripping voltammograms on $Pd_{ML}Pt(111)$ after CO_2 reduction at different vertex potentials in CO_2-saturated pH 3 with 1 mM $KClO_4$ electrolyte, measured at 10 mV s^{-1}.

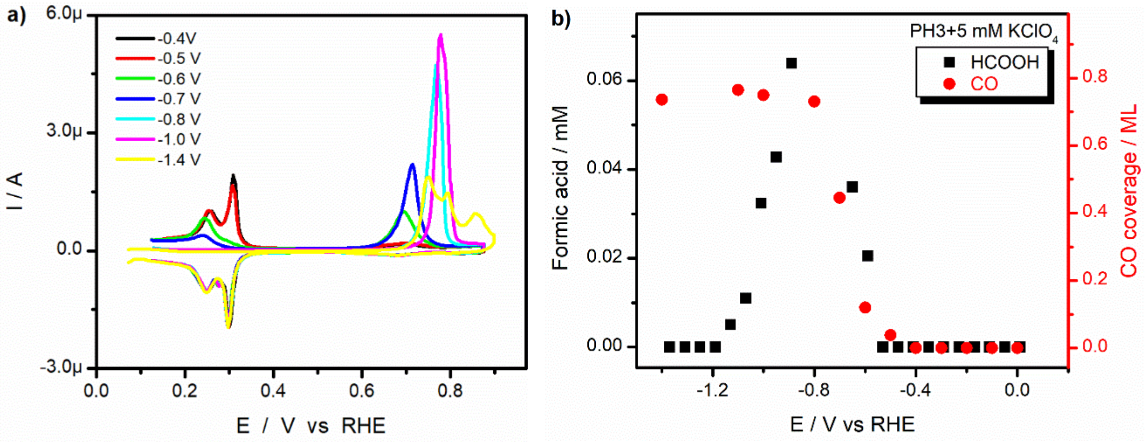

Figure S5 a. CO stripping voltammograms on $Pd_{ML}Pt(111)$ after CO_2 reduction at different vertex potentials in CO_2-saturated pH 3 with 5 mM $KClO_4$ electrolyte, measured at 10 mV s^{-1}. **b.** HCOOH formation and the calculated CO coverage as a function of potential.

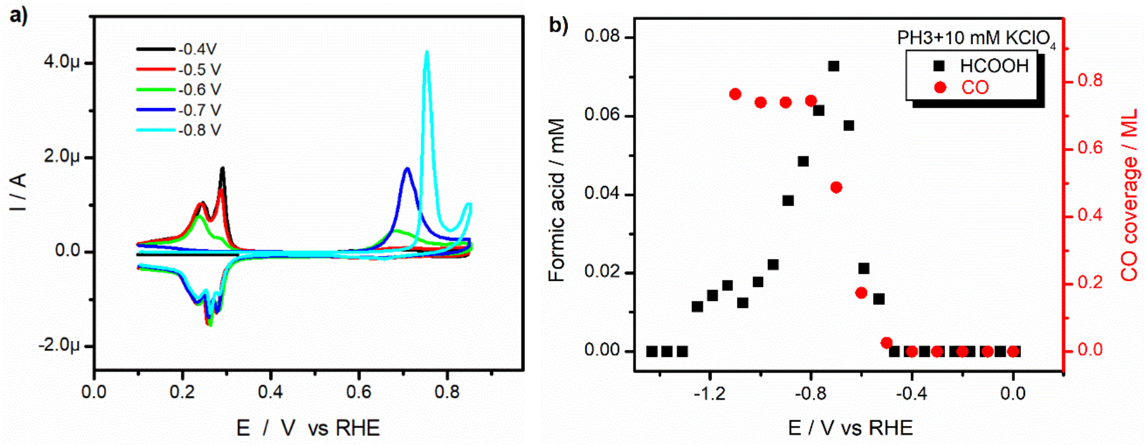

Figure S6 a. CO stripping voltammograms on $Pd_{ML}Pt(111)$ after CO_2 reduction at different vertex potentials in CO_2-saturated pH 3 with 10 mM $KClO_4$ electrolyte, measured at 10 mV s^{-1}. **b.** HCOOH formation and the calculated CO coverage as a function of potential.

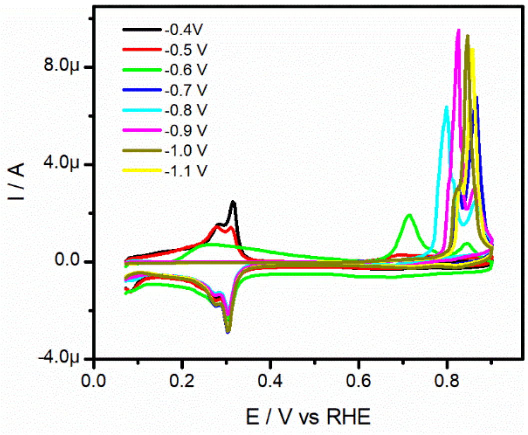

Figure S7. CO stripping voltammograms on $Pd_{ML}Pt(111)$ after CO_2 reduction at different vertex potentials in CO_2-saturated pH 3 with 99 mM $KClO_4$ electrolyte, measured at 10 mV s^{-1}.

Figures S3a, S4, S5a, S6a and S7 show oxidative stripping voltammograms of adsorbed CO obtained during CO_2RR by increasing the vertex potential in steps of 0.1 V from –0.4 V_{RHE}. With the positive going scan, decreasing peaks (peak at 0.31 V_{RHE} decreasing first) in the potential region between +0.05 and +0.35 V_{RHE} suggest adsorbed CO. CO_{ads} oxidation peaks are observed in the potential region between +0.65 and +0.9 V_{RHE}, the peaks varying under different conditions. These different CO oxidation peaks may result from different *CO coverage or changes of local electrolyte after CO_2RR. After oxidation of *CO, the typical CV features in pH 3 electrolyte are again observed in the negative going scan. In some voltammograms, CV features of $Pd_{ML}Pt(111)$ slightly deviate from the standard, which might be due to minor loss of Pd atoms during CO_2RR. Moreover, peaks in the potential region between +0.05 and +0.35 V_{RHE} decreases with increasing of *CO obtained from CO_2RR, which leads to more CO oxidation current in potential region between +0.65 and +0.9 V_{RHE} and corresponding higher CO coverage.

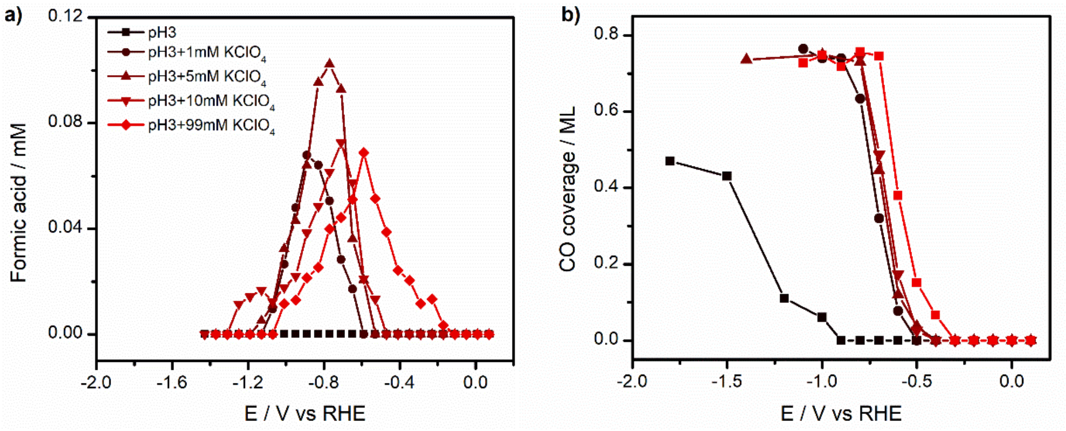

Figure S8. A summary of CO and formic acid generation during CO_2RR in CO_2 saturated pH 3 electrolytes in the presence of 0, 1, 5, 10, 99 mM $KClO_4$.

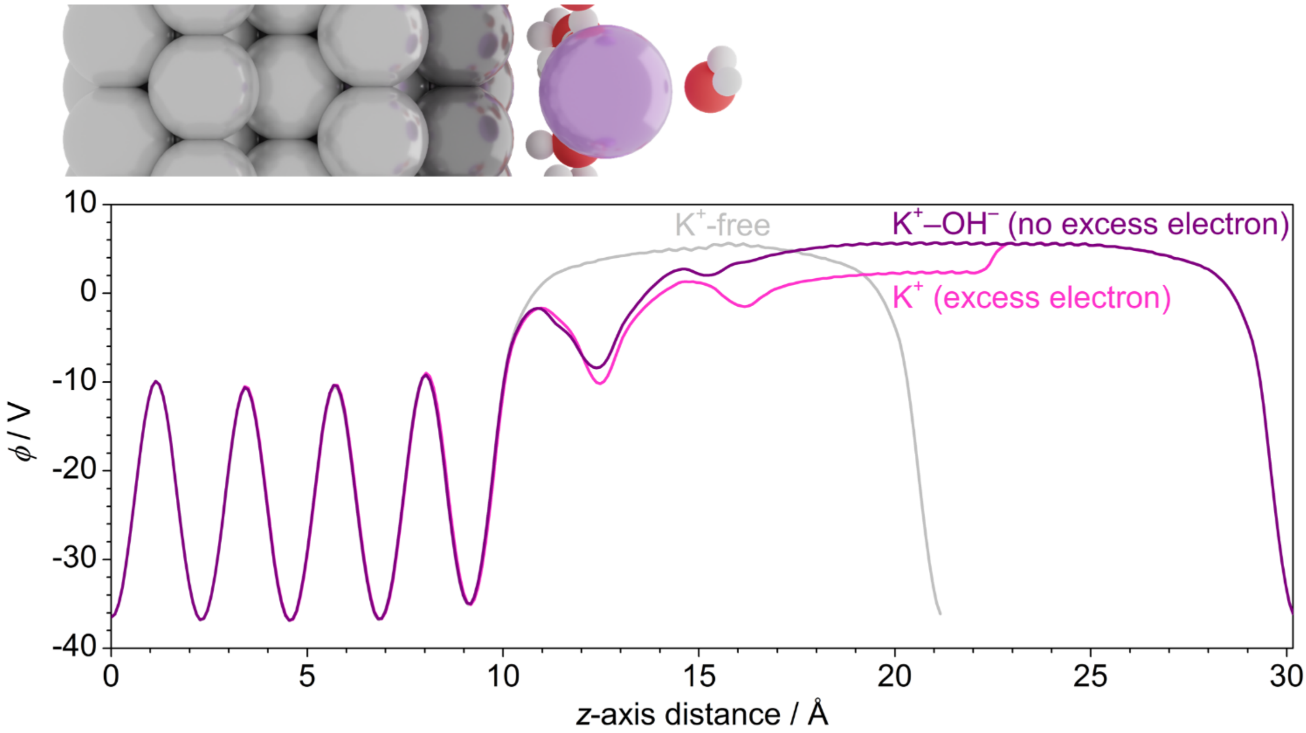

Figure S9. Electrostatic potential (Φ) profile across the simulation cell (*z*-direction, reported on top) for the cation-free (gray line) and near-cation ($d_{K+\text{-surf}}$ = 4 Å) systems with and without excess electron (magenta and purple line respectively).

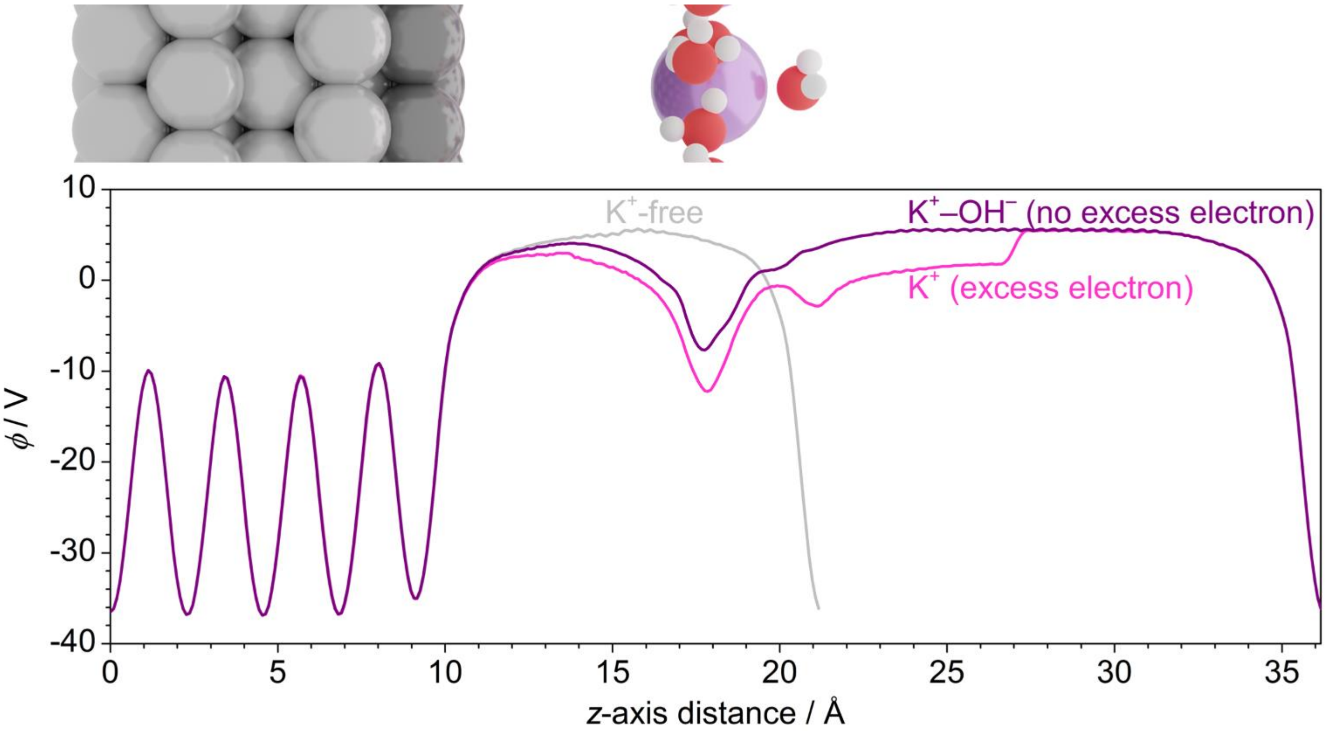

Figure S10. Electrostatic potential (Φ) profile across the simulation cell (z-direction, reported on top) for the cation-free (gray line) and far-cation ($d_{K+\text{-surf}}$ = 9 Å) systems with and without excess electron (magenta and purple line respectively).

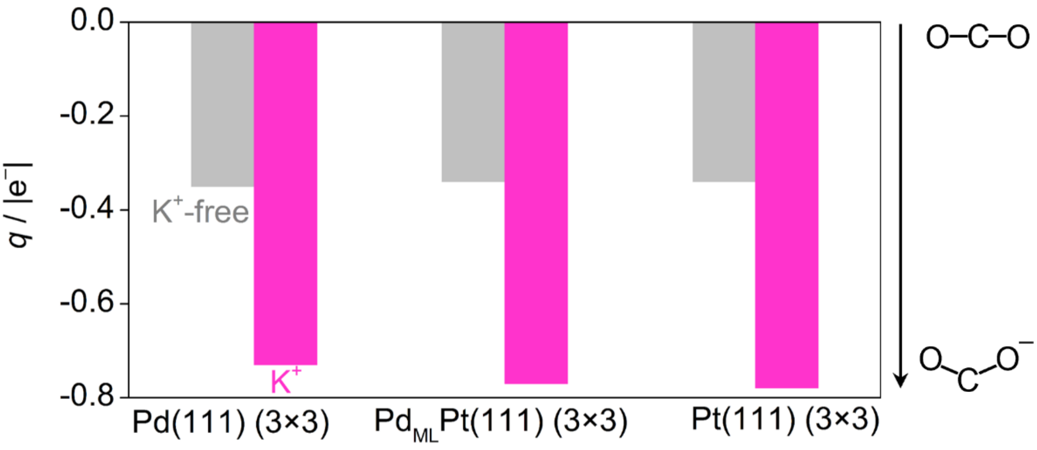

Figure S11. Bader charges for adsorbed CO_2^- on Pd(111) (3×3), Pd_{ML}Pt(111) (3×3), and Pt(111) (3×3). The gray column reports the baseline case for the adsorbate without K^+, while the near-K^+ system (with excess electron) is indicated in magenta. Solvated K^+ was here simulated with three water molecules within its coordination shell.

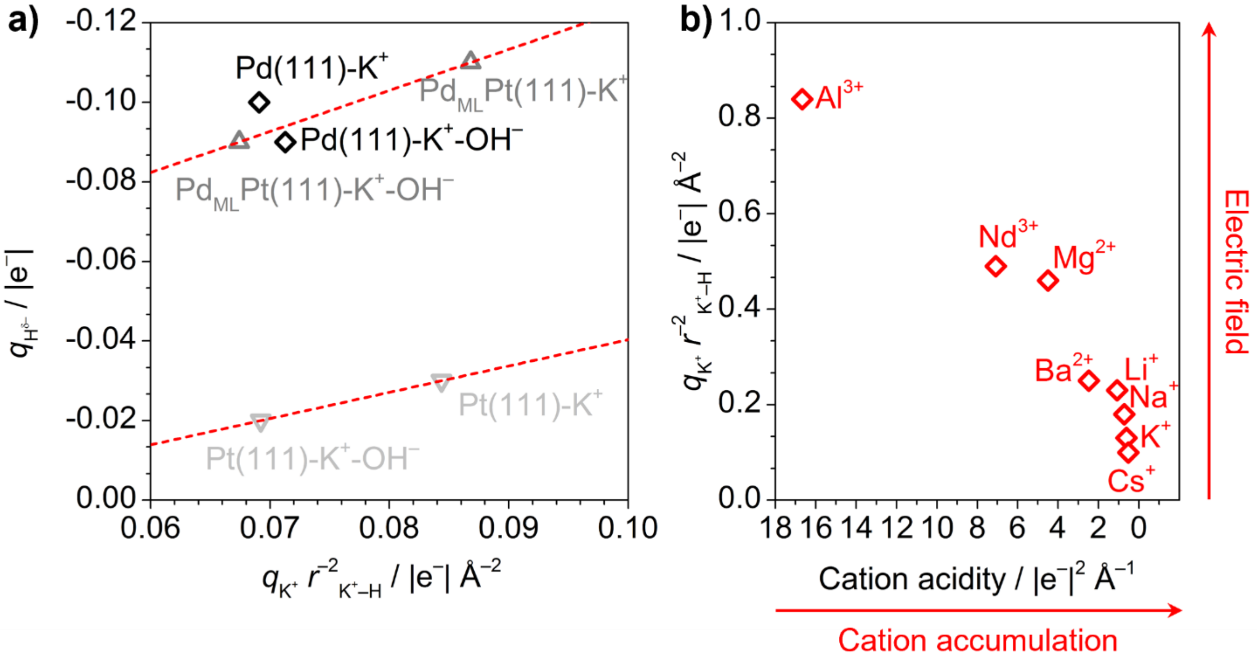

Figure S12. **a**. Correlation between $H^{\delta-}$ Bader charges and cation-induced electric field, proportional to $q^{K+}/r^2_{K+\text{-}H}$. **b**. Cation-induced electric field vs cation acidity for alkali, bi-valent, and tri-valent cations. Cation acidity affects cation accumulation, while electric field favors the formation of hydrides on the surface. The distance between cation and hydrogen is here taken as the state-of-the-art cation-water distance reported for solvated cations (see Table S3).[5-6]

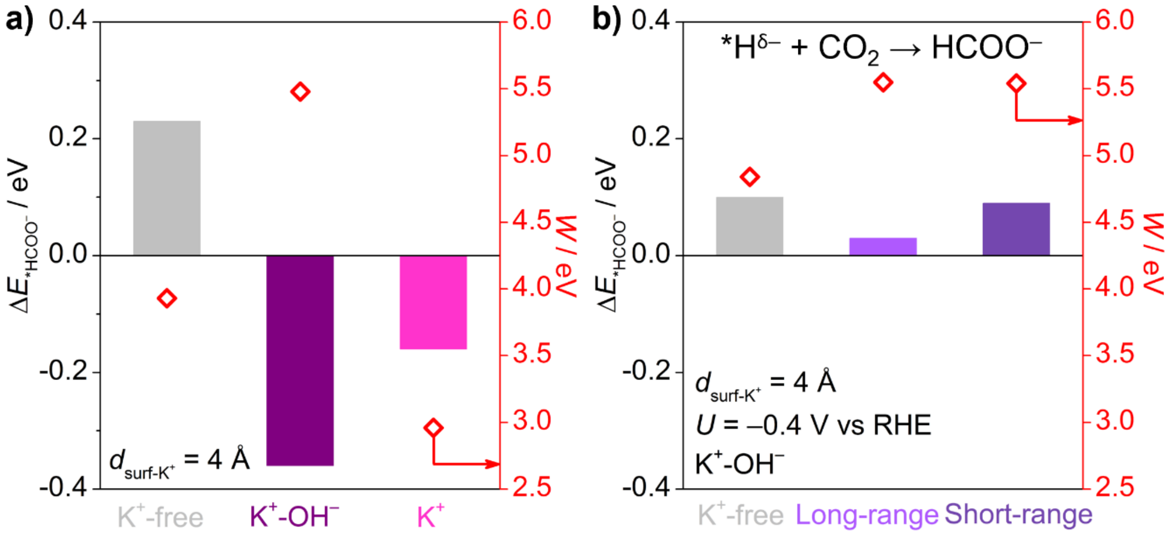

Figure S13. a. DFT energy on $Pd_{ML}Pt(111)$ (3×3) relative to cation-induced outer-sphere CO_2 activation to $HCOO^-$ for the K^+-free (gray) and near-K^+ with and without balancing OH^- (purple and magenta) for different values of metal work functions (right *y*-axis). **b.** DFT energy relative to the $HCOO^-$ formation step on $Pd_{ML}Pt(111)$ (3×3) supercell for cation-free (gray), far-K^+ (light purple), and near-K^+ (dark purple) cases at U = –0.4 V vs RHE. Values of metal work function for clean surfaces are given at the right *y*-axis. For details on the model, see Figure 5a-b and Computational Details.

Table S1. Calculated work function for the $Pd_{ML}Pt(111)$ (3×3) surface for clean surface and $*CO_2$ for the K^+-free, the near-K^+ ($d_{K+\text{-}surf}$ = 4 Å), and far-K^+ ($d_{K+\text{-}surf}$ = 9 Å) cases, with and without balancing OH^-. Cation and H_2O molecules (within bracket) were absent in the K^+-free case.

System	W / eV				
	K^+-free	Near K^+	Near K^+ + OH^-	Far K^+	Near K^+ + OH^-
Clean surface	+4.84	+2.35	+5.54	+1.74	+5.55
$*CO_2^-$ (+ K^+ + $5H_2O$)	+5.05	+5.09	+5.59	+5.14	+5.41

Table S2. Calculated Bader charges of the $Pd_{ML}Pt(111)$ (3×3) surface for clean surface and $*CO_2$ for the K^+-free, the near-K^+ ($d_{K+\text{-}surf}$ = 4 Å), and far-K^+ ($d_{K+\text{-}surf}$ = 9 Å) cases, with and without balancing OH^-. Cation and H_2O molecules (within brackets) were absent in the K^+-free case.

System	q_{surf} / $\|e^-\|$				
	K^+-free	Near-K^+	Near K^+ + OH^-	Far K^+	Near K^+ + OH^-
Clean surface	+0.00	–0.62	–0.24	–0.05	–0.05
$*CO_2^-$ (+ K^+ + $5H_2O$)	+0.35	–0.07	+0.29	+0.30	+0.30

Table S3. Properties of alkali, bi-valent, and tri-valent cations, taken from Refs. [5-8].

Cation	q_{cat} / $\|e^-\|$	ionic radius / Å	$d_{cat\text{-}O}$ / Å	Acidity / $\|e^-\|^2$ $Å^{-1}$	$q_{cat}/r^2_{cat\text{-}O}$ / $\|e^-\|$ $Å^{-2}$
Li^+	1	0.92	2.08	1.09	0.23
Na^+	1	1.39	2.36	0.72	0.18
K^+	1	1.64	2.80	0.61	0.13
Cs^+	1	1.88	3.14	0.53	0.10
Mg^{2+}	2	0.89	2.09	4.49	0.46
Ba^{2+}	2	1.61	2.81	2.48	0.25
Al^{3+}	3	0.54	1.89	16.67	0.84
Nd^{3+}	3	1.27	2.47	7.09	0.49

References

1. McCrum, I. T.; Koper, M. T. M., The role of adsorbed hydroxide in hydrogen evolution reaction kinetics on modified platinum. *Nature Energy* **2020,** *5* (11), 891-899.
2. Monteiro, M. C.; Dattila, F.; Hagedoorn, B.; García-Muelas, R.; López, N.; Koper, M., Absence of CO_2 electroreduction on copper, gold and silver electrodes without metal cations in solution. *Nat. Catal.* **2021,** *4* (8), 654-662.
3. Chan, K.; Nørskov, J. K., Potential Dependence of Electrochemical Barriers from ab Initio Calculations. *J. Phys. Chem. Lett.* **2016,** *7* (9), 1686-1690.
4. Chan, K.; Nørskov, J. K., Electrochemical Barriers Made Simple. *J. Phys. Chem. Lett.* **2015,** *6* (14), 2663-2668.
5. Monteiro, M. C. O.; Dattila, F.; Lopez, N.; Koper, M. T. M., The Role of Cation Acidity on the Competition between Hydrogen Evolution and CO2 Reduction on Gold Electrodes. *J. Am. Chem. Soc.* **2022,** *144* (4), 1589-1602.
6. Marcus, Y., Ionic radii in aqueous solutions. *Chem. Rev.* **1988,** *88* (8), 1475-1498.
7. Haynes, W. M., *CRC handbook of chemistry and physics*. CRC press: 2016.
8. Waegele, M. M.; Gunathunge, C. M.; Li, J.; Li, X., How cations affect the electric double layer and the rates and selectivity of electrocatalytic processes. *J. Chem. Phys.* **2019,** *151* (16), 160902.
